# Physical fitness, serum relaxin and duration of gestation

**DOI:** 10.1186/s12884-015-0607-z

**Published:** 2015-08-14

**Authors:** Eva Thorell, Laura Goldsmith, Gerson Weiss, Per Kristiansson

**Affiliations:** Department of Public Health and Caring Sciences, Uppsala University, Uppsala, SE-75122 Sweden; Department of Obstetrics, Gynecology and Women’s Health, New Jersey Medical School of Rutgers University, Newark, NJ 07103 USA

## Abstract

**Background:**

Women are recommended to perform regular exercise during pregnancy but the impact of physical fitness on duration of gestation and miscarriage is inconsistent. In addition, a dose-response relation between the amount of weekly exercise and increased risk of miscarriage in early pregnancy has been observed. Previous studies have mostly used an epidemiologic method. Larger studies using careful measurement of physical fitness are needed. Besides physical fitness, maternal circulating concentrations of the hormone relaxin have been associated with decreased duration of gestation.

**Methods:**

A prospective cohort including 20 women with miscarriage and 460 women with spontaneous onset of labour, recruited from maternal health care centres in central Sweden, were examined in early pregnancy regarding estimated absolute peak oxygen uptake ($\dot{V}{O}_2$_peak, est._) by cycle ergometer test, and maternal circulating serum relaxin concentrations.

**Results:**

Women with miscarriage displayed the highest level of absolute $\dot{V}{O}_2$_peak, est._ (2.61 l/min) and the lowest serum relaxin concentrations (640 ng/l). Among women with spontaneous onset of labour, the mean absolute $\dot{V}{O}_2$_peak, est._ increased successively from the lowest estimated oxygen uptake of 2.31 l/min among those with preterm birth (*n* = 28), to an oxygen uptake of 2.49 l/min among women with postterm birth (*n* = 31). An opposite trend was shown regarding serum relaxin concentrations from women with miscarriage to those with postterm birth. Serum relaxin concentrations, but not absolute $\dot{V}{O}_2$_peak, est._ was significantly and independently associated with duration of gestation in women with miscarriages, and absolute $\dot{V}{O}_2$_peak, est._, age and multiple pregnancy were independently associated with duration of gestation in women with spontaneous onset of labour.

**Conclusions:**

Physical fitness appears to be a protective factor of established pregnancies and not significantly involved in the risk of early miscarriage. Additional studies are needed to more clearly define the role of relaxin in miscarriage.

## Background

Women who exercise regularly in the non-pregnant state frequently continue to do so during pregnancy [1], while the proportion of women who exercise declines as pregnancy progresses [2]. It is recommended that women perform regular exercise during pregnancy [3], although the impact of physical fitness on duration of gestation or miscarriage is inconsistent. A positive association between physical fitness and duration of gestation at delivery has been suggested in some studies [4–6] and disputed by others [7–10]. A dose-response relation between the amount of weekly exercise and the risk of miscarriage in early pregnancy has been observed [11], whereas high intensity exercise among healthy female athletes was shown not to compromise foetal growth and development in established pregnancies [12, 13].

The polypeptide hormone relaxin has been linked to duration of gestation at child birth [14] and miscarriage in early pregnancy [15, 16]. Relaxin is a pleiotrophic peptide hormone of the insulin-like growth factor family, and it has been known to be a pregnancy related hormone for 80 years. Relaxin is involved in regulation of biochemical processes in remodelling the extracellular matrix of cervix and vagina during pregnancy and relaxin receptors have been found in fibroblasts in the cervix [17, 18]. Relaxin also has growth effects on the uterus and placenta, influences vascular development and proliferation in the endometrium, and causes biochemical changes needed for rupture of the foetal membranes at term [19]. Increased expression of endogenous decidual relaxin is seen in women with preterm rupture of membranes [20]. In very early pregnancy, there is an initial increase of maternal serum concentrations of relaxin until a peak at about the 12^th^ gestational week followed by a decline until about the seventeenth week. Thereafter, serum concentrations remain stable for the duration of the pregnancy [21].

To make a thorough analysis of the impact of physical fitness and serum relaxin concentrations in early pregnancy on duration of gestation, careful measurement of physical fitness, a reliable serum relaxin analysis and accurate information about the time of end of pregnancy are required. In the present study, we used estimated absolute peak oxygen uptake ($\dot{V}{O}_2$_peak, est._) assessed in early pregnancy as a measure of physical fitness, in addition to well established, validated assay for serum relaxin analyses, and accurate information about the timing and circumstances of miscarriage and delivery. Estimated absolute peak oxygen uptake is suggested to be an adequate method to be used also in pregnancy as a reliable indicator of physical fitness [2, 22–24]. The objective of the study was to analyse the impact of physical fitness and relaxin on duration of gestation of the general population.

## Methods

### Study design

A prospective longitudinal cohort study.

### Setting

Pregnant women in Sweden have the right to attend a Maternal Health Center free of charge. The centres are operated by the County Councils, or are subcontracted to the Councils. The centres in this particular region are staffed by general practitioners, midwives and administrative staff, and they all follow the same general procedure with repeated appointments during pregnancy, and one appointment postpartum.

### Study population

**Fig. 1:**
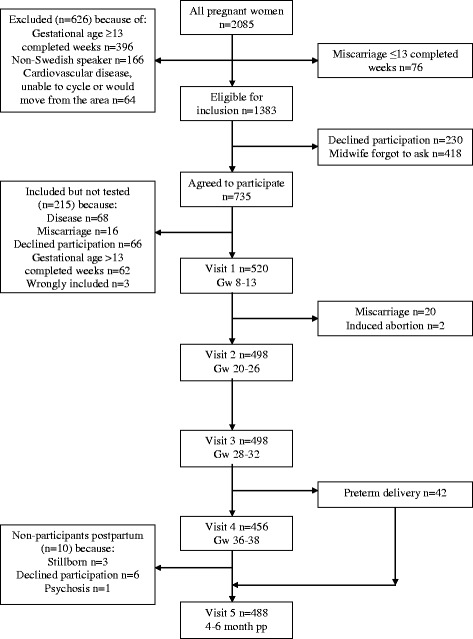
Flow chart of participants and non-participants throughout the study

All women in early pregnancy who attended eight maternal health centers in the city of Örebro (population 128,000), and two each in the municipalities Kumla and Hallsberg (populations 20,000 and 15,000) close to Örebro, between March 2001 and June 2003, were identified, a total of 2,085 women. Of these women, 418 women were not invited to participate since the degree of commitments varied across the maternal health care centres and 932 women either declined participation in the study, or were excluded for various reasons (described in Fig. 1), leaving 735 women to participate. Of these, 215 did not take the cycle ergometer test, leaving a study population of 520 women. All women gave written informed consent to participate in the study. Inclusion criterion was duration of gestation less than 13 completed weeks and exclusion criteria were non-Swedish speaker, cardiovascular disease or on-going treatment of hypertension.

The duration of gestation was confirmed by ultrasound examination in the estimated gestational week 17 and registered as completed weeks of gestation. Information about the onset of the delivery and multiple pregnancies were retrieved from the medical records of the respective obstetric centre. The duration of gestation was categorised as miscarriage <22 completed weeks (<154 days), preterm birth <37 completed weeks (154–258 days), birth at term 37 - <42 completed weeks (259–293 days) and postterm birth ≥42 weeks (≥294 days).

### Data collection

The baseline data collection was performed on average at 10.9 (C.I. 10.8–11.1) weeks of gestation. The women were asked to complete a questionnaire indicating the number of previous pregnancies and deliveries, cigarette smoking habits and education. Height without shoes (measured to the nearest centimetre with a wall-mounted tape measure) and weight with indoor clothing without shoes (measured by a lever balance, in kilograms to one decimal place) was recorded.

Physical fitness was estimated once at baseline using the submaximal cycle ergometer heart rate method [25]. Absolute estimated peak oxygen uptake ($\dot{V}{O}_2$_peak, est._) was measured. This test has been described in detail in a previous report [2].

Peripheral blood was sampled once in early pregnancy, in average at 9.1 (C.I. 8.2–10.0) gestational weeks. The blood samples were centrifuged immediately after sampling and the serum was stored at -20 °C until analysis.

### Relaxin assay

Concentrations of relaxin in each serum sample were determined using a homologous, human relaxin-specific radioimmunoassay previously described [26, 27]. The intraassay and the interassay coefficients of variation were 8.9 % (*n* = 12 observations) and 9.8 % (*n* = 14 assays) respectively. All serum samples were analysed blindly in duplicate. The lowest and highest detectable relaxin values were 80 and 3450 ng/l, respectively. Five samples with values below the lowest detectable assay point and four samples with values above the highest assay point were replaced by 80 and 3450 ng/l.

### Statistical analysis

Statistical analyses were performed using SAS software, version 9.3 (SAS Institute Inc., Cary, NC, USA). Associations between continuous and ordinal data were tested using Pearson’s and Spearman’s correlation coefficients, respectively. Serum relaxin concentrations, absolute $\dot{V}{O}_2$_peak, est._ and duration of gestation were normally distributed among women with miscarriage but not among women with spontaneous onset of labour. Because there was a big enough sample size the central limit theorem was applied and the assumption of normality satisfied and the mean with 95 % confidence intervals (C.I.) were calculated. For regression analyses the General Linear Model was used. Several simple linear regression analyses with duration of gestation as the dependent variable and each of the possible determinants as independent variables were performed. The significant variables in the simple regression analyses were entered into a multiple linear regression analysis to determine the adjusted associations of absolute $\dot{V}{O}_2$_peak, est._ and serum relaxin concentrations on duration of gestation. I.e., the difference of serum relaxin concentrations between women with single and multiple pregnancies was taking care of statistically. The scale assigned for categorization of ordinal factors used in the model as independent variables was no/yes for current cigarette smoking or quit cigarette smoking for less than six months ago, no/yes for completed university studies and no/yes for multiple births. No multicollinearity problem was found (1.03 ≤ variance inflation factor ≤ 1.06). Only two-tailed tests were used. The level of significance in the simple regression analyses was set at *p* < 0.1 and in the other analyses at *p* < 0.05.

The Research Ethics Committee of Örebro University, Sweden, approved the study in 2001 and supplemented in 2003 (reference number 217/01).

## Results

Characteristics in early pregnancy of women with miscarriage and delivery preterm, term and postterm are shown in Table 1. The highest level of absolute $\dot{V}{O}_2$_peak, est._ was displayed among women with miscarriage (2.61 l/min) who also displayed the lowest serum relaxin concentrations (640 ng/l). Among the 460 women with spontaneous onset of labour the absolute $\dot{V}{O}_2$_peak, est._ increased successively from the lowest oxygen uptake of 2.31 l/min among those with preterm birth to an oxygen uptake of 2.49 l/min among women with postterm birth. An opposite trend was shown regarding serum relaxin concentrations from women with miscarriage to those with postterm birth.

**Table 1:** Characteristics of women with miscarriage, spontaneous delivery: preterm, term and postterm

	Miscarriage (<141 days)		Preterm birth (141-158 days)		Term birth (159–293 days)		Postterm birth (>293 days)	
Characteristic	n	Mean (C.I.)/Proportion (%)	n	Mean (C.I.)/Proportion (%)	n	Mean (C.I.)/Proportion (%)	n	Mean (C.I.)/Proportion (%)
Age (yr)	20	30.4 (27.9–32.8)	28	28.8 (27.1–30.5)	401	28.7 (28.3–29.1)	31	30.5 (28.7–32.4)
Weight (kg)	20	68.1 (63.8–72.5)	28	69.2 (64.1–74.3)	401	67.8 (66.6–69.0)	31	68.6 (62.6–74.6)
Height (m)	20	1.66 (1.63–1.68)	28	1.65 (1.63–1.67)	401	1.67 (1.66–1.67)	31	1.66 (1.63–1.68)
No previous pregnancy	3	15	10	35.7	176	43.9	15	48.4
No previous delivery	5	25	11	39.3	206	51.4	19	61.3
> one previous delivery	4	20	1	3.6	47	11.7	3	9.7
Education, completed university studies	8	40	10	35.7	185	46.1	12	38.7
Current smoker or quit before 6 months ago	3	15	6	21.4	78	19.4	3	9.7
Absolute $\dot{V}{O}_2$_peak, est._ (l/min)	20	2.61 (2.35–2.89)	28	2.31 (2.15–2.47)	401	2.44 (2.39–2.48)	31	2.49 (2.28–2.69)
Serum relaxin (ng/l)	20	640 (511–768)	28	792 (565–1020)	392	767 (728–806)	31	687 (598–776)
Duration of gestation (days)	20	98 (88–108)	28	247 (244–250)	401	280 (279–281)	31	297 (296–297)
Multiple pregnancy	-	-	4	14.3	2	0.5	0	-
Caesarean section	-	-	3	10.7	20	5.0	7	22.6

$\dot{V}{O}_2$_peak,. est._ = estimated peak oxygen uptake

Among the 520 women, 20 women had spontaneous miscarriage (range 5 to 22 completed gestational weeks), 460 had spontaneous onset of labour. Among the remaining 40, 2 had induced abortion before gestational week 20, 23 did not go into labour and were delivered by elective caesarean section, and 15 had induced delivery before 42 weeks of pregnancy. Among the 460 women with spontaneous onset of labour 386 delivered vaginally (non-instrumentally), 44 delivered with assistance of vacuum extraction or forceps, and 30 were delivered by Caesarean section.

Among the 20 women with miscarriage the mean duration of gestation was 98 days (C.I. 88–108) which was significantly different (*p* < 0.0001) from the mean time of blood sampling at 66 days of gestation (C.I. 60–72).

### Correlation between determinants measured in early pregnancy and duration of gestation

**Fig. 2:**
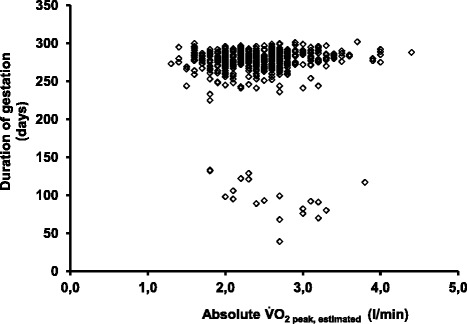
Duration of gestation by absolute $\dot{V}{O}_2$_peak, est._. Duration of gestation among 20 women with miscarriage and 460 women with spontaneous onset of labour by absolute $\dot{V}{O}_2$_peak, est._

Absolute $\dot{V}{O}_2$_peak, est._ was inversely correlated to duration of gestation among women with miscarriage (*r* = -0.52, *p* = 0.02) and positively to duration of gestation among women with spontaneous onset of labour (*r* = 0.12, *p* = 0.01), Fig. 2.

**Fig. 3:**
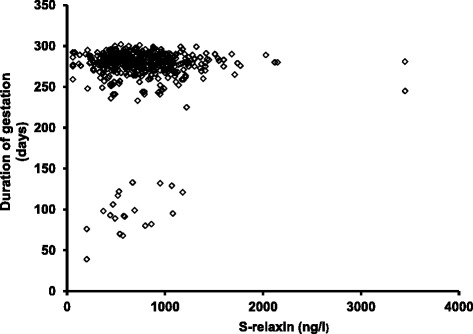
Duration of gestation by serum relaxin concentrations. Duration of gestation, among 20 women with miscarriage and 460 women with spontaneous onset of labour, by serum relaxin concentrations

Serum concentrations of relaxin showed a positive association to duration of gestation among women with miscarriage (*r* = 0.48, *p* = 0.03) but no association to duration of gestation among women with spontaneous onset of labour (*r* = 0.07, *p* = 0.11), Fig. 3. A similar result was observed when the outliers were removed from the analysis.

### Regression analyses

Among women with miscarriage absolute $\dot{V}{O}_2$_peak, est._ was inversely and serum relaxin positively associated with duration of gestation in simple regression analyses but none of the other measured factors, Table 2. In a multiple regression analysis with the significant variables included in the model, serum relaxin remained independently associated with duration of gestation. The R^2^ of the model was 0.47 (<0.0001).

**Table 2:** Association of factors measured in early pregnancy on duration of gestation among women with spontaneous delivery (preterm, term or postterm)

	Simple linear regression			Multiple linear regression R^2^ = 0.07, *p* < 0.0001	
Variable	Estimate	R^2^	*p*=	Estimate	*p*=
Age (yr)	0.26	0.00	0.05	0.20	0.12
Weight (kg)	0.04	0.00	0.33		
Height (m)	12.1	0.00	0.22		
Number of deliveries	−0.53	0.00	0.48		
Number of pregnancies	−0.30	0.00	0.56		
University education (no/yes)	0.01	0.00	0.98		
Current smoking (no/yes)	−0.80	0.00	0.40		
Absolute $\dot{V}{O}_2$_peak, est._ (l/min)	3.2	0.02	0.004	2.33	0.04
Serum relaxin (ng/l)	0.00	0.00	0.11	0.00	0.48
Multiple pregnancy (no/yes)	−25.2	0.06	<0.0001	−24.5	<0.0001

$\dot{V}{O}_2$_peak,. est._ = estimated peak oxygen uptake

Among women with spontaneous onset of labour absolute $\dot{V}{O}_2$_peak, est._ and age were positively and multiple pregnancies was inversely associated to duration of gestation but none of the other measured factors, in simple linear regression analyses, Table 3. In a multiple regression analysis, with the significant variables included, multiple pregnancy and absolute $\dot{V}{O}_2$_peak, est._ remained significantly and independently associated to duration of gestation. The R^2^ of the model was 0.07 (*p* < 0.0001).

**Table 3:** Association of factors measured in early pregnancy on duration of gestation among women with miscarriage

	Simpe linear regression			Multiple linear regression R^2^ = 0.47, *p* < 0.0001	
Variable	Estimate	R^2^	*p*=	Estimate	*p*=
Age (yr)	−1.4	0.09	0.21		
Weight (kg)	−0.5	0.04	0.41		
Height (m)	−154	0.13	0.12		
Number of deliveries	−4.8	0.05	0.35		
Number of pregnancies	−4.0	0.05	0.32		
University education (no/yes)	3.1	0.03	0.46		
Current smoking (no/yes)	0.30	0.00	0.97		
Absolute $\dot{V}{O}_2$_peak, est._ (l/min)	−20.5	0.22	0.04	−15.7	0.09
Serum relaxin (ng/l)	0.05	0.28	0.02	0.04	0.04

$\dot{V}{O}_2$_peak,. est._ = estimated peak oxygen uptake

## Discussion

Increased physical fitness was associated with increased duration of gestation among women with spontaneous onset of labour and decreased duration of gestation among women with miscarriage although the latter not significant. Low concentrations of serum relaxin was shown among women with miscarriage whereas within this group increased serum relaxin concentrations were associated with increased duration of gestation. The results might have clinical implications regarding physical activity and risk of miscarriage in later pregnancy.

The strengths of the present study were the prospective approach, use of validated methods and the number of participants who performed the cycle ergometer test in early pregnancy. An additional cycle ergometer test during pregnancy might have added to a small proportion since the difference between the oxygen uptake in early pregnancy and postpartum was small [2]. Use of a direct method of oxygen uptake would have further increased the accuracy of the physical fitness assessment but this would have been difficult to realise.

To the best of our knowledge no previous study has investigated the influence of physical fitness assessed by absolute $\dot{V}{O}_2$_peak, est._ on duration of gestation among women who miscarried or women who gave birth.

An increased risk of miscarriage among women with increased level of physical exercise has been shown in previous epidemiologic studies [11, 28–30]. A similar trend was shown in the present study of physical fitness although not significant and without information on early miscarriages. In a previous experimental study no increase of miscarriages was found across fit women with different load of physical exercise during pregnancy [28].

A suggested protective effect of physical fitness on preterm births and an increased proportion of post-term birth, particularly by exercise between gestational week 17 and 30 has been reported [10]. This is supported by the results of the present study and others [4–6], and disputed by others [7–9].

Low serum relaxin concentrations in early pregnancy have been associated with increased risk of miscarriage [15, 16], which was shown also in the present study. However, among women with miscarriage those with higher relaxin concentration had a longer duration of gestation, in the present study. With an otherwise viable foetus this might enable relaxin treatment to save an imminent miscarriage, especially when considering that serum relaxin is known to have an important role in implantation by remodelling and immunotolerance [31].

## Conclusions

Physical fitness appears to be a protective factor of established pregnancies and not significantly involved in the risk of early miscarriage. Increased serum relaxin concentration to avoid imminent miscarriage with a viable foetus is suggested a future research challenge.

## Abbreviations

$\dot{V}{O}_2$_peak, est_: Estimated peak oxygen uptake
C.I.: Confidence interval
